# Two-Color Spatially Resolved Tuning of Polymer-Coated Metasurfaces

**DOI:** 10.1021/acsnano.3c11760

**Published:** 2024-01-30

**Authors:** Sarah L. Walden, Purushottam Poudel, Chengjun Zou, Katsuya Tanaka, Pallabi Paul, Adriana Szeghalmi, Thomas Siefke, Thomas Pertsch, Felix H. Schacher, Isabelle Staude

**Affiliations:** † Institute of Solid State Physics, Abbe Center of Photonics, Friedrich Schiller University Jena, Helmholtzweg 3, 07743 Jena, Germany; ‡ Institute of Applied Physics, Abbe Center of Photonics, Friedrich Schiller University Jena, Albert-Einstein-Strasse 15, 07745 Jena, Germany; § Institute of Organic Chemistry and Macromolecular Chemistry, Friedrich Schiller University Jena, Lessingstr. 8, 07743 Jena, Germany; ∥ Jena Center for Soft Matter (JCSM), Friedrich Schiller University Jena, Philosophenweg 7, 07743 Jena, Germany; ⊥ Center for Energy and Environmental Chemistry (CEEC), Friedrich Schiller University Jena, Philosophenweg 7, 07743 Jena, Germany; # Institute of Microelectronics, Chinese Academy of Sciences, Beitucheng West Road 3, 100029 Beijing, People’s Republic of China; ∇ Fraunhofer Institute for Applied Optics and Precision Engineering, Albert-Einstein-Str. 7, 07745 Jena, Germany

**Keywords:** nanophotonics, dielectric metasurfaces, photoswitches, tunable metasurfaces, spiropyran, azobenzene

## Abstract

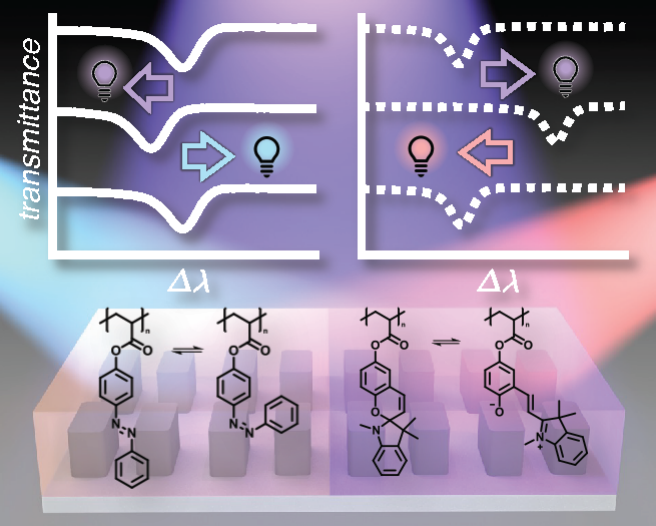

For the realization of truly reconfigurable metasurface technologies, dynamic spatial tuning of the metasurface resonance is required. Here we report the use of organic photoswitches as a means for the light-induced spatial tuning of metasurface resonances. Coating of a dielectric metasurface, hosting high-quality-factor resonances, with a spiropyran (SPA)-containing polymer enabled dynamic resonance tuning up to 4 times the resonance full-width at half-maximum with arbitrary spatial precision. A major benefit of employing photoswitches is the broad toolbox of chromophores available and the unique optical properties of each. In particular, SPA and azobenzene (AZO) photoswitches can both be switched with UV light but exhibit opposite refractive index changes. When applied to the metasurface, SPA induced a red shift in the metasurface resonance with a figure of merit of 97 RIU^–1^, while AZO caused a blue shift in the resonance with an even greater sensitivity of 100 RIU^–1^. Critically, SPA and AZO can be individually recovered with red and blue light, respectively. To exploit this advantage, we coated a dielectric metasurface with spatially offset SPA- and AZO-containing polymers to demonstrate wavelength-dependent, spatially resolved control over the metasurface resonance tuning.

Optical metasurfaces have revolutionized the ability to control and manipulate EM fields. By carefully designing a 2D array of subwavelength nanoresonators, often referred to as meta-atoms, the light–matter interactions can be engineered to precisely control the scattering properties of the incident field.^1−3^ Nanoscale control over the light propagation has proved crucial for the development of ultrathin lenses,^4^ polarization convertors,^5^ beam shapers,^6^ holographic displays,^7^ and sensors.^8^ The vast majority of fabricated metasurfaces are designed to display specific properties or functionalities and cannot be altered after fabrication. However, for emerging metasurface applications such as optical switching, dynamic displays, and arbitrary wavefront generators, static metasurfaces need to be replaced with dynamic, tunable implementations.^9−11^ There is a plethora of ways in which metasurface tuning can be achieved. Most reports to date employ a single electrical,^12−15^ optical,^16−18^ mechanical,^19−21^ thermal,^22−24^ or chemical^25,26^ stimulus to tune the optical resonances.

When considering the mechanisms available for metasurface resonance tuning, stimuli-responsive polymer coatings emerge as an attractive option, owing to the broad scope for tailored design and ability to efficiently alter the refractive index surrounding the nanoresonators.^27^ Driven heavily by the need for sustainability and biocompatibility, the last decades have seen an explosion in the breadth of polymers designed to respond to a particular stimulus, such as temperature, pH, electrical potential, mechanical stress, or light.^28−30^ Light is a particularly attractive stimulus, as it affords spatiotemporal control, that is, arbitrary spatial control over where the stimulus is applied, and temporal control over the rate the beam can be modulated for rapidly responding materials. Light also provides an additional level of selectivity by virtue of the wavelength. The library of photochromophores is vast, but in particular photoswitches stand out as an advantageous approach for optical tuning, as they can rapidly transition between two isomers upon the stimulus of light and are recoverable either optically or thermally in the dark.^31,32^ To date, azobenzene photoswitches have been the most broadly applied material for nano-optic applications,^33,34^ as they possess the attractive features of rapid switching within seconds, high photostability across hundreds of cycles, and unique photomorphic behavior.^35−38^ In particular, for metasurface tuning, Ren et al. coated a plasmonic metasurface with azobenzene-containing polymer films to tune the polarization of transmitted NIR light using a low-intensity green laser.^39^

As the challenges and future applications of metasurface technologies become more complex, so too must the devices become able to address these challenges. Additional functionalities can be incorporated into metasurface devices by exploiting multiple stimuli. Recently, our group demonstrated increased functionality in metasurface resonance tuning by imbedding a Si metasurface in a liquid crystal cell and tuning the liquid crystal orientation with the application of voltage and heat.^40^ We subsequently demonstrated the tuning of quasi-bound states in the continuum (quasi-BIC)^41^ using the dual stimulus of light and temperature.^42^ To this end, Si metasurfaces were coated with polymers containing light-responsive azobenzene and temperature-responsive triethylene glycol molecules. Application of either UV light or heat would induce a 1 or 3 nm resonance shift, respectively. However, much larger resonance shifts up to 5 nm could be achieved when both stimuli were applied simultaneously, providing an extra level of control.

To date, none of the works realizing tunable metasurfaces based on hybridization with light-responsive polymers has fully exploited the unique advantages offered by using light as a stimulus, namely, spatiotemporal control in combination with wavelength-selected control. Here we close this gap by applying a thin film of a spiropyran-containing polymer to reversibly tune the quasi-BIC resonance of a Si metasurface by using UV and visible LEDs. By patterning the optical stimulus, we demonstrate spatially resolved resonance tuning down to a resolution of 11 μm. Further to this, we exploit wavelength control by spatially patterning spiropyran-containing polymers and azobenzene-containing polymers,^42^ to demonstrate spatially resolved, wavelength-dependent metasurface resonance tuning.

## Results and Discussion

### Metasurface Design and Fabrication

**Figure 1:**
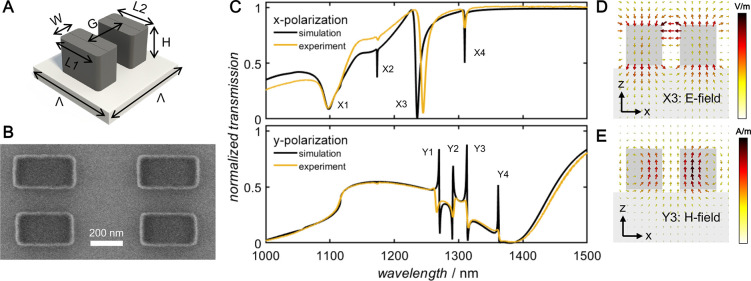
Metasurface design and characterization. (A) Schematic diagram of the metasurface unit cell comprising two bars of differing length. (B) SEM image of the fabricated metasurface corresponding to an EBL exposure dose of 100 μC cm^–2^. (C) Simulated (black) and measured (yellow) transmission measurements of the fabricated metasurface shown in (B) for (top) *x*- and (bottom) *y*-polarized incident light, (D) Electric field profiles of the in-plane electric dipole resonance X3 and (E) magnetic field distribution of the antiparallel, out-of-plane magnetic dipole resonance Y3.

For sensitive tuning applications, high-quality-factor (*Q*-factor) resonances, such as those produced from BICs, are desired. BICs are nonradiating modes within a broad frequency spectrum that become confined within the photonic structure.^43^ In theory, they manifest as an infinitely narrow dip in the transmission spectrum. Perfect BICs are not attainable in reality; however, quasi-BIC resonances can be engineered by introducing asymmetries into the nanoresonator array.^41^ For this work asymmetric silicon metasurface arrays were designed to feature high-*Q*-factor quasi-BIC resonances in the NIR, enabling applications in the telecommunications wavelength range including optical logic operations or diffractive neural networks. The unit cell, schematically pictured in Figure 1A, has a period of Λ = 770 nm and comprises two silicon nanoresonators separated by a center-to-center distance of *G* = 360 nm. The two bars are designed to possess equal height and width but differing lengths and host quasi-BIC resonances with near-field profiles primarily located in the gaps between the resonators. This design is intended to maximize the sensitivity to changes in the polymers. Full fabrication details are provided in Section 1.1 of the Supporting Information. Briefly, amorphous silicon thin films were prepared and coated with a conductive chromium layer. Electron beam lithography (EBL) using a negative tone electron-beam resist was then employed to fabricate the 600 μm × 600 μm 2 bar metasurface arrays at 8 different EBL doses, ranging from 70 to 140 μC cm^–2^. Varying the EBL dose produced multiple arrays featuring a systematic variation of the nanoresonator width and length. After EBL, reactive ion etching removed silicon in the unexposed areas, leaving only the rectangular nanoresonators. A scanning electron micrograph (SEM) of the structures fabricated with an EBL dose of 100 μC cm^–2^ is provided in Figure 1b. Effective dimensions of the fabricated structures were determined by comparing the measured spectra to spectra simulated in the commercial software package CST Studio Suite and were found to be *L*_1_ = 405 nm, *L*_2_ = 373 nm, *W* = 242 nm, and *H* = 270 nm. The measured and simulated transmission spectra are listed in Figure 1C.

The differing lengths of the nanoresonators, and gaps between them, introduce several asymmetries into the design, leading to narrow resonances in the NIR wavelength region. Fabrication tolerances meant that differences are observed in the resonance widths and positions of the fabricated structures. A full discussion of the electromagnetic resonances has been provided previously;^42^ for brevity only the resonances relevant to this work will be discussed here. For *x*-polarized incident light, a sharp, narrow dip with a *Q*-factor of 141 is observed at λ_X3_ = 1235 nm. The simulated near-field profile in Figure 1D shows that this resonance corresponds to an antiparallel out-of-plane electric dipole resonance (X3). This resonance produces strong field enhancements between the two nanoresonators of the unit cell, making it particularly sensitive to environmental changes. Experimentally, the transmission dip is observed slightly red-shifted at λ = 1245 nm. Under *y*-polarized excitation, four sharp Fano resonances arising from the breaks in symmetry are observed. They spectrally overlap a broad transmission dip arising from the magnetic dipole resonance. Of these sharp peaks, the antiparallel, out of plane magnetic dipole resonance at λ_Y3_ = 1313 nm depicted in Figure 1E will be most relevant to this work.

### Spiropyran Coated Metasurfaces

To introduce tunability into the system, the metasurface was spin coated with a thin layer of a light-responsive, spiropyran-containing polymer (pSPA). The homopolymer of spiropyran acrylate, pSPA, was synthesized via free-radical polymerization initiated with 2,2-azobis(isobutyronitrile) (AIBN). Full synthetic details can be found in Section 2 of the Supporting Information. The synthesized polymer was characterized by using a combination of size exclusion chromatography (SEC) and nuclear magnetic resonance spectroscopy (NMR). The SEC analysis of pSPA revealed a molar mass (*M*_n_) of 5500 g/mol with a rather broad dispersity of 1.9.

**Figure 2:**
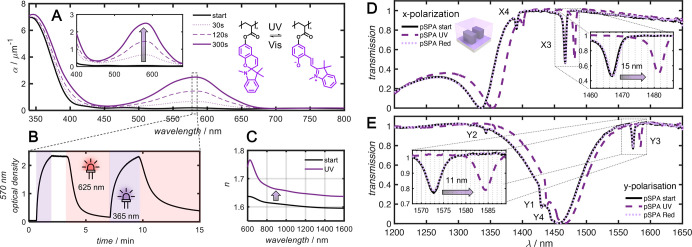
Spiropyran photoswitch characterization. (A) Absorption measurements of a 675 nm thick film of pSPA on a glass coverslip measured at regular intervals during UV irradiation. Insets show a zoomed-in view of visible absorption peak and structure of pSPA before and after isomerization. (B) Kinetic optical density measurements at 570 nm during irradiation with UV and red light. (C) Refractive index of 675 nm thick pSPA film determined by ellipsometry before and after UV irradiation. (D) Measured NIR transmission of the pSPA-coated metasurface for *x*-polarized incident light before irradiation (solid black line), after UV irradiation (dashed purple line), and after 625 nm red light irradiation (dotted light purple line). Note that the dotted light purple line overlaps the solid black line, indicating complete reversibility. The insets show a close-up of the high-*Q* resonances. (E) same as (D) but with *y*-polarized light.

The absorption properties of pSPA during switching were monitored by using UV–visible spectroscopy (UV–vis). For this a thin film of pSPA was prepared on a glass coverslip using the spin-coating procedure outlined in Polymer Film Preparation at 1500 rpm, producing a film thickness of 675 nm. The absorption spectrum of the film was measured during irradiation with a 340 nm LED delivering 12 μW cm^–2^ to the film surface and is presented in Figure 2A. UV irradiation induced isomerization of the visibly transparent spiropyran to the dark purple merocyanine isomer, evidenced by the broad absorption peak spanning 450–670 nm. To better gauge the speed of the switching, *in situ* UV–vis measurements were performed using the custom-built apparatus described in In situ UV-Visible Spectroscopy. Kinetic optical density measurements of the film at 570 nm, corresponding to the visible absorption peak, were recorded every 250 ms and are presented in Figure 2B. Irradiation with a 365 nm LED delivering 10 mW cm^–2^ led to a rapid increase in the 570 nm optical density, indicative of spiropyran switching. Complete switching was observed within 2 min. Subsequent irradiation with a 625 nm LED delivering 85 mW cm^–2^ induced reverse isomerization, recovering the transparent spiropyran isomer within 5 min. Repeated irradiation cycles were found to introduce fatigue into the system, limiting the full recovery of spiropyran. This fatigue can result from a variety of factors, including the accumulation of defects in the molecular structure or photodegradation. The dispersion of a pSPA film prepared on a silica substrate is presented in Figure 2C as obtained by spectroscopic ellipsometry analysis. Comparison of the measurements before and after UV irradiation revealed a significant increase in refractive index across the NIR wavelength region as a result of isomerization.

With the optical properties of pSPA characterized, pSPA films were spin-coated onto the metasurface using the procedure at 1500 rpm. Initial trials indicated that the film thicknesses produced using 1500 rpm resulted in slow, weak shifts in the metasurface resonances (see Section 3.5 in the Supporting Information for details). It was therefore decided to modify the spin-coating procedure to 3000 rpm, which reduced the pSPA film thickness to 475 nm and resulted in an improved light penetration through the polymer film. Transmission measurements of the pSPA-coated metasurface (pSPA-MS) are summarized in Figure 2D,E. As a first observation, they reveal that the application of the polymer film caused a shift in the electric dipole resonance X3 to 1467 nm and a marked increase in *Q*-factor to 407. The magnetic dipole resonance visible in the transmission measurements with *y*-polarized light also underwent a significant shift to 1573 nm. Application of UV light to pSPA-MS induced a further red shift in these resonances by 15 and 11 nm for the X3 and Y3 resonances, respectively. The achieved sensitivity, defined by the shift of the resonance peak divided by the change in the refractive index unit (RIU), is 348 nm RIU^–1^ corresponding to a figure of merit (FOM), defined by the ratio of the sensitivity to the full-width at half-maximum (FWHM) of the resonance of 97 RIU^–1^. This constitutes a shift of the X3 resonance over 4 times the FWHM and is comparable to other systems hosting quasi-BIC resonances.^44−47^ The red shift in resonances is attributed to the increase in refractive index of the pSPA polymer upon UV irradiation. This is particularly significant for the X3 resonance, which is located between the nanoresonators, where the polymer film is situated. Subsequent irradiation with 625 nm irradiation recovered the original resonance position. The observed shifts were verified by performing simulations of the polymer-coated metasurface before and after UV irradiation, assuming polymer thicknesses of 500 nm and refractive indices obtained from ellipsometry measurements (refer to Section 3.2 of the Supporting Information). The simulations predicted a 15 nm red shift in resonance X3 for *x*-polarized light, and a 20 nm red shift in the Y3 resonance for *y*-polarized incident light. The minor discrepancy between simulation and experiment is attributed to the polymers not uniformly filling the gaps between the nanoresonators. Improved penetration of the polymer solution throughout the metasurfaces might be achieved in a flow cell structure.^48^

**Figure 3:**
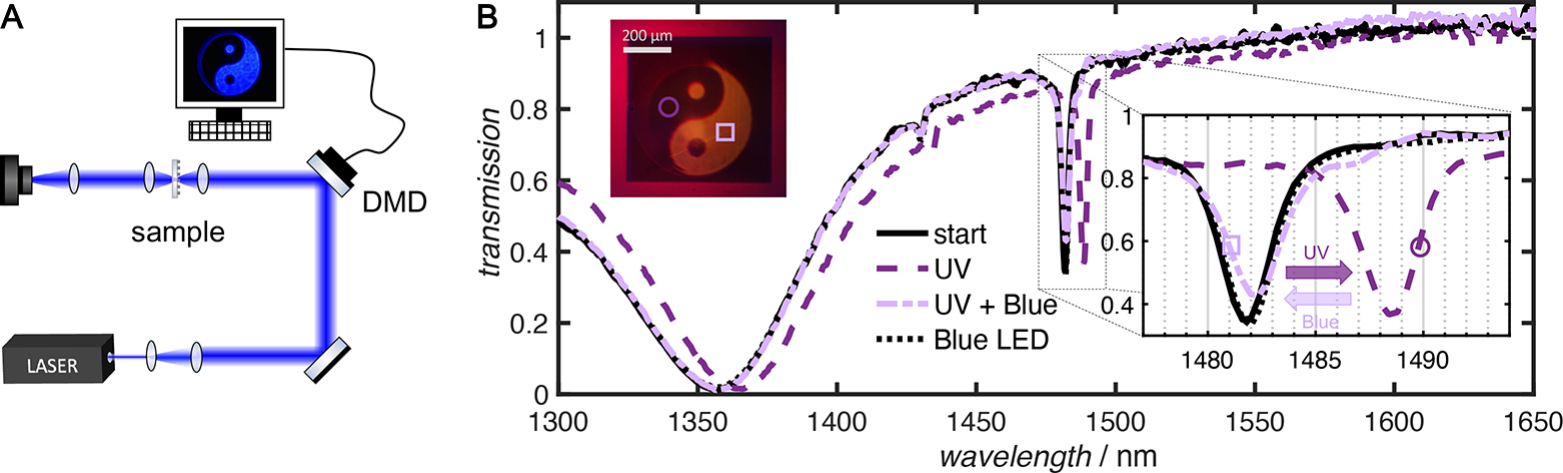
Spatially resolved metasurface tuning. (A) Schematic diagram of the experimental apparatus. (B) Measured transmission of the pSPA-coated metasurface prior to irradiation (solid black line), in the areas exposed to UV light only (dashed purple line), to UV and blue light (dot-dashed light purple line), and finally after the entire sample was exposed to 450 nm irradiation (dotted black line). Note that the dashed black line overlaps the solid black line, indicating complete reversibility. The incident light is polarized along the *x*-direction. The inset in (B) shows a zoomed-in view of resonance X3.

Next, spatial tuning of the metasurface resonances was demonstrated by employing a digital micromirror device (DMD) to spatially pattern 450 nm laser irradiation onto the hybrid metasurface. These results are summarized in Figure 3. A home-built optical setup, described in more detail in Spatial Patterning of Polymer-Coated Metasurface, was employed to project the image generated by the DMD onto the metasurface sample (see Figure 3A). Note that a fresh coating of 475 nm thick pSPA was applied in these experiments. First, the sample was exposed to UV light to switch the spiropyran to the open merocynanine form and shift the metasurface resonance to longer wavelengths (see purple dashed spectrum in Figure 3B). The metasurface array was then exposed to 450 nm irradiation and patterned into the shape of a yin-yang symbol to recover the spiropyran isomer in the irradiated regions. Owing to the visible color difference between the isomers, the irradiated areas within the metasurface array can be clearly identified in a white light image (inset of Figure 3B). Spectra measured prior to UV irradiation (Figure 3B, solid black line), in the UV-exposed areas (Figure 3B, dashed purple line), and in the UV- and blue-exposed areas (Figure 3B, dashed-dotted light purple line) demonstrate the spatial control over the metasurface tuning. Finally, exposing the entire area to a 450 nm LED recovered the initial metasurface resonance position (Figure 3B, dotted black line). Using the current optical setup, spatial patterning down to a resolution of 11 μm was achieved (Section 3.4 in the Supporting Information). However, a prime advantage of using photoresponsive polymers to tune metasurface resonances is that the spatial resolution of the resonance tuning is solely limited by the resolution of the light field.

### Two-Color Tuning with Spiropyran- and Azobenzene-Containing Polymers

The strength of using photoswitches for metasurface resonance tuning lies in the broad toolbox of photoswitches available for such a task. The wavelength degree of freedom afforded with light stimulus provides the opportunity to combine multiple photoswitches to achieve even more heightened control over the metasurface resonance switching. We have already shown in this work that UV irradiation of spiropyran-coated metasurfaces induces a red shift in the metasurface resonance. Previous works have employed azobenzene-containing polymers and reported a blue shift in the metasurface resonance until UV irradiation.^42^ The combination of these two photoswitches enables spatially resolved red or blue shifting of the metasurface resonance, controlled solely by the wavelength of irradiation.

**Figure 4:**
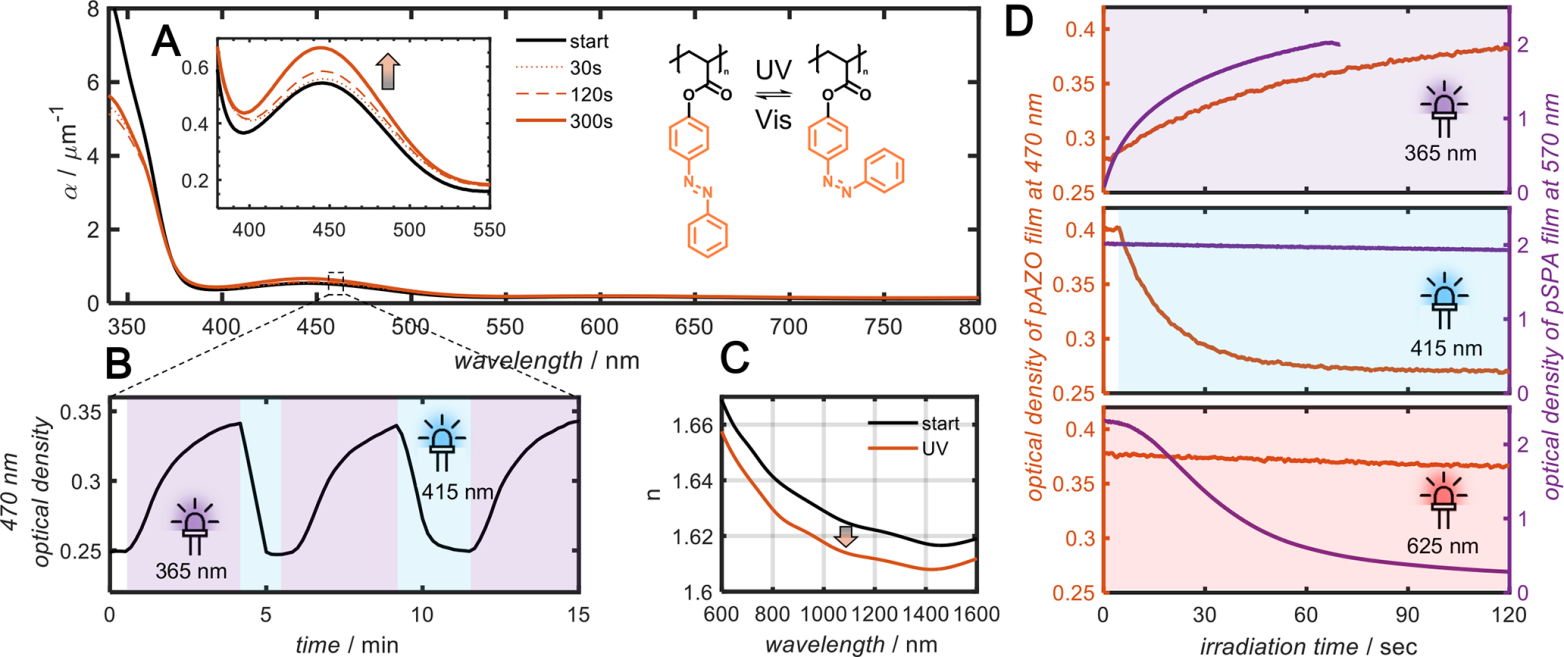
Optical characterization of azobenzene thin films. (A) Absorption measurements of a 689 nm thick film of pAZO measured at regular time intervals during UV irradiation. Insets show a zoomed-in view of the visible absorption peak and structure of pAZO before and after isomerization. (B) Kinetic optical density measurement of a pAZO film at 470 nm during irradiation with UV and blue light. (C) Refractive index measurements of a 568 nm thick pAZO film before and after UV irradiation. (C) Kinetic optical density measured of pAZO (left axis) and pSPA (right axis) films during irradiation with (top) UV light, (middle) red light, and (bottom) blue light, demonstrating the independent addressability of the two photoswitches.

The first step in achieving such control over metasurface tuning is to identify optical regions where the two photoswitches, spiropyran and azobenzene, can be independently accessed. This is nontrivial, since both photoswitches absorb UV light and isomerize to a visible-light-absorbing isomer. The optical properties of spiropyran during switching have already been discussed in detail, but we briefly consider the optical properties of azobenzene. Polymers containing azobenzene pendant groups (pAZO) were prepared using a previously discussed synthetic route.^42^ A 689 nm thick film of pAZO was prepared on a glass cover slide by using the previously described spin-coating procedure at 1500 rpm . The absorption spectrum of the film measured at regular intervals during 340 nm irradiation is presented in Figure 4A. The thermodynamically favored *trans* isomer of azobenzene exhibits a strong UV absorption band, as well as a weak, visible absorption peak centered at around 450 nm. A minor increase in visible light peak is observed upon irradiation, indicative of azobenzene isomerization. The rate of isomerization was investigated using the *in situ* UV–vis setup. The kinetic optical density of the film at 470 nm (offset slightly from the absorption maxima to avoid fluorescence) is presented in Figure 4B. Irradiation with 10 mW cm^–2^ of 365 nm light led to almost complete isomerization within 5 min. Subsequent irradiation with blue light (λ_max_ = 415 nm, 186 mW cm^–2^) recovered the original isomer within 30 s. In contrast to pSPA, UV irradiation of pAZO led to a reduction in the refractive index of the polymer film, as determined from ellipsometry measurements of the pAZO film prepared on silica substrates, depicted in Figure 4C (refer to Section 1.2 of the Supporting Information for the full spectrum). The switching between the two isomers can be repeated over many cycles, without affecting its photostability.^42^

Despite both azobenzene and spiropyran switching under UV light, the spectral separation between their visible absorption bands affords narrow wavelength regions where the two photoswitches can be addressed independently. The wavelength-dependent switching behavior is summarized in Figure 4D. As a first step, thin films of pSPA and pAZO were prepared on separate glass coverslips according to the previously outlined procedure and their absorption spectra were recorded at regular intervals during irradiation with several different colored LEDs. Kinetic tracking of the optical density at the visible absorption peak is presented in Figure 4D. First, both films were exposed to UV light (λ_max_ = 365 nm, 10 mW cm^–2^) and, as expected, an increase in the visible absorption peak was observed for both photoswitches (Figure 4D, top panel). Upon irradiation with blue light (λ_max_ = 415 nm, 186 mW cm^–2^), the visible band of the pAZO sample rapidly decreased (Figure 4D, middle panel), while no change was observed in the pSPA spectrum. Subsequent irradiation with UV light once again recovered the *cis*-azobenzene isomer. Finally, both samples were exposed to red LED light (λ_max_ = 625 nm), leading to a rapid decrease in the visible peak of pSPA, with only minor reduction in the pAZO spectrum due to thermal reversion (Figure 4D, lower panel). Irradiation with a 450 nm LED led to a rapid reduction in the visible peak for both films.

**Figure 5:**
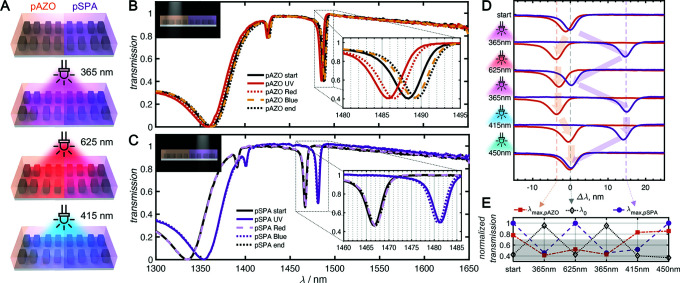
Two-color polymer-coated metasurface tuning. (A) Schematic diagram of spatially patterned polymer-coated metasurface, showing irradiation of the whole sample with various colored LEDs. (B, C) Transmission measurements of *x*-polarized light incident on (B) metasurface array fabricated with EBL dose 70 μC cm^–2^ coated with pSPA-MS and (C) metasurface array fabricated with EBL dose 120 μC cm^–2^ coated with pAZO-MS measured under different irradiation conditions. Note that the starting spectrum overlaps with the final spectrum, indicating complete reversibility. (D) Shifts in metasurface resonances under irradiation with various LEDs showing the selective recovery of pSPA-MS and pAZO-MS shifts with red and blue light irradiation, respectively. (E) Tracking the transmission at the initial resonance wavelength (λ_0_), wavelength of maximum shift of pSPA-MS (λ_max,pSPA_) and wavelength of maximum shift of pAZO-MS (λ_max,pAZO_).

Once the activation windows had been identified, it was possible to incorporate the two polymers into a single metasurface array. Initially, the two polymers were mixed together in a 1:1 ratio and spin-coated onto the metasurface, and the resonance tuning was tested. However, interactions between the two photoswitches undermined the ability to independently address the switching behavior after spin coating (for full details refer to Section 3.6 of the Supporting Information). To avoid these interactions, it was decided to sequentially spin coat the metasurface arrays using a procedure detailed in Polymer Film Preparation. First, half the metasurface arrays (EBL doses 70–100 μC cm^–2^) were coated with a 500 nm thick layer of pSPA (pSPA-MS). Then, the second half of the metasurface arrays (EBL doses 110–140 μC cm^–2^) were coated with a 570 nm thick layer of pAZO (pAZO-MS). The spatially separated polymer coatings are depicted in Figure 5A. It is important to mention that due to practical limitations with the spin-coating procedure, it was not feasible to apply both coatings to a single metasurface array. The variation in EBL dose, along with the slight variations in film thickness and refractive index, led to slightly different resonance wavelengths for the pAZO-MS and pSPA-MS before the application of any stimulus. To simplify comparison of the metasurface resonance tuning, the resonances are presented both as the raw measured spectra (λ, Figure 5B,C) as well as a relative shift from the original resonance position (Δλ, Figure 5D,E).

First, the sample was preirradiated with 450 nm light for 30 s, and the transmission of pAZO-MS and pSPA-MS was measured (Figure 5B, solid black). Next the sample was irradiated with UV light (λ_max_ = 365 nm) for 2 min, inducing a 4 nm blue shift in the pAZO-MS (Δλ = −4 nm) and a huge 15 nm red shift in pSPA-MS (Δλ = 15 nm). Remarkably, the sensitivity and FOM for pAZO-MS are 441 nm RIU^–1^ and 100 RIU^–1^, respectively, greatly exceeding that found for pSPA-MS. To independently reverse the pSPA tuning, red light (λ_max_ = 625 nm) was applied for 5 min, leaving the pAZO resonance unaffected, while shifting the pSPA-MS resonance back to the original position at λ_pSPA_ = 1467 nm. The sample was once again exposed to UV irradiation for 2 min before blue light (λ_max_ = 415 nm) was applied for 1 min to selectively reverse the pAZO-MS resonance slightly past the original position to λ_pSPA_ = 1489 nm, while leaving the pSPA-MS resonance unaffected. Final irradiation with a 450 nm LED recovered both resonances back to their original positions. The small difference between the initial and final positions of the pAZO-MS resonance is due to the time used for preirradiation with 450 nm. A longer preirradiation time removes this discrepancy. It is important to note that the switching times depend heavily on the incident light intensity. Here focused LEDs were employed to induce isomerization, resulting in switching times ranging from a few seconds to several minutes. These times could be significantly reduced with the use of coherent light sources that can be focused much more tightly, achieving higher intensities. One must do so with caution, however, as excess UV irradiation can accelerate potential oxidation reactions and photodegradation, especially in case of SPA-based materials.^49^ From a chemical perspective, the switching speed could be further improved by increasing the photoswitch incorporation into the polymer; however, this introduces significant synthetic and solubility challenges. In the prepared state, the polymer-coated metasurfaces were stable for several weeks, with the lifetime primarily being limited by temperature, humidity, oxidation, and photodegradation. Encapsulation of the device to isolate the polymers from the surrounding environment can be used to further prolong the lifetime. However, proper storage conditions and environmental controls are crucial to ensuring their long-term performance and reliability.

The independent addressability of the two polymer coatings provides an interesting avenue for information coding, programmable optical devices, logical processing,^50^ or trainable models for diffractive optical neural networks using optically tuned metasurface transmission.^51,52^ Purely with the application of spatially patterned light, the transmission values at discrete wavelengths can controlled to either a HIGH or LOW state, where a transmission of 0.7 is considered the threshold value (above the gray shaded area in Figure 5E). Based on this processing criteria, initially the value at Δλ_0_ is LOW and Δλ_pSPA_ and Δλ_pAZO_ are HIGH. UV light inverts these positions. The combination of UV and red or blue light selectively activates Δλ_pSPA_ or Δλ_pAZO_ alone to the HIGH position, producing a wavelength-dependent logical processor.

## Conclusion

Spatial variability plays a key role in the metasurface functionality. Applications like wavefront manipulation rely on high-resolution spatial encoding. For the future realization of fully programmable metasurface devices, spatial control over the resonance tuning is essential. Herein we have demonstrated the use of two different photoswitch-containing polymers as an attractive method for optically tuning metasurface resonances. By illuminating a spiropyran-coated metasurface with spatially patterned light, we were able to demonstrate reversible spatial control over the metasurface quasi-BIC resonance wavelength. We subsequently exploited the disparate resonance shifts in spiropyran- and azobenzene-coated metasurfaces to demonstrate wavelength-dependent, spatially resolved resonance tuning. We propose that this platform could have applications in information coding, logical processing, or training of optical diffractive neural networks.

## Experimental Methods

### Metasurface Fabrication

The silicon metasurfaces were fabricated by using an electron beam lithography procedure in combination with reactive ion etching. Initially, commercially available amorphous silicon thin films situated on a glass substrate (Tafelmaier Dünnschicht Technik GmbH) were etched to the target silicon thickness of 270–280 nm by argon-ion beam etching (Oxford Instruments Plasma Technology, Ionfab 300) and covered with a conductive chromium layer by means of ion beam sputter deposition (Oxford Instruments Plasma Technology Ionfab 300). A negative electron-beam resist (100 nm EN038, Tokyo Ohka Kogyo Co., Ltd.) was then spin-coated onto the sample and exposed by a variable-shaped electron-beam lithography system (Vistec SB 350OS) to define the areas to be selectively dissolved in a developer (OPD 4262) for 30 s. After the exposed pattern was transferred to the chromium layer by ion-beam etching (Oxford Instruments Plasma Technology, Ionfab 300LC), the silicon layer was etched through the chromium mask by reactive ion etching (RIE-ICP, Sen-tech SI-500 C). Finally, the residual resist and chromium mask were effectively eliminated through a thorough washing process. Acetone was used to remove the resist, while a ceric ammonium nitrate based solution, applied within a megasonic bath at 50 °C, successfully eliminated the chromium mask.

### Polymer Film Preparation

Metasurface samples were first plasma cleaned using an O_2_ plasma at 100 W for 5 min. Stock solutions of the pSPA (5 wt % in CHCl3) and pAZO (5 wt % in THF) polymers were prepared and filtered over a 0.22 μm pore size polytetrafluoroethylene (PTFE) syringe filter. Prior to spin coating, the stock solutions were heated to 80 °C for 5 min. 40 μL of stock solution was drop-cast onto the glass slide or metasurface, and initially a spin-coat procedure of 1500 rpm (acceleration of 500 rpm/s) for 5 min was used. When these films were found to be too thick, the spin rate was increased to 3000 rpm (acceleration of 1000 rpm/s) for 5 min. The resulting film thicknesses are outlined in the Section 1.1 in the Supporting Information.

Where both polymers were applied to a single metasurface, the pAZO thin film was prepared first by drop-casting 20 μL of stock solution to half the metasurface arrays and applying the previously mentioned spin-coat procedure at 3000 rpm. 20 uL of pSPA stock solution was then drop-cast onto the remaining metasurface arrays, and the same spin-coat procedure was applied. The viscosity of the stock solutions meant that the layers remained separated. For more details refer to Section 1.1 of the Supporting Information.

### Ellipsometry

Synthesized solutions of pSPA and pAZO were spin-coated onto Si substrates at a speed of 1500 rpm for 5 min, resulting in coating layer thicknesses from around 150 to 450 nm, depending on the corresponding solution viscosity. Next, the samples were characterized by ellipsometry (J.A. Woollam M-2000) covering a wavelength range from 245 to 1670 nm. To determine the refractive indices of the polymers in their different phases, an in situ light stimulus was applied. The measured ellipsometry data were processed with the program CompleteEASE assuming a B-Spline absorbing layer. The dispersive refractive indices are provided in Figure S2 of the Supporting Information.

### Metasurface Transmission Measurements

The polarization-dependent transmittance spectra of the metasurfaces were measured using a custom-built optical setup depicted in Figure S3 of the Supporting Information. The white light source was a tungsten halogen lamp (Thorlabs SLS301), fiber coupled and collimated with a condenser lens (*f* = 7 mm). The light was then transmitted through a polarizer and was incident on the sample. The transmitted light propagated through two apertures in the real and Fourier planes to limit the measurement area and incident angles, respectively. A removable mirror was used to select the imaging path, where the light was directed to a CMOS camera (Thorlabs DCC1645C-HQ) for sample alignment. When the mirror was removed, the light was coupled into a fiber using a condenser lens (*f* = 20 mm) and directed to a Yokogawa AQ6370D optical spectrum analyzer to record the spectra at near-infrared wavelengths. For light-responsive tuning experiments, the illuminating LEDs (blue or UV) were positioned adjacent to the beam path and focused onto the sample using a 25 mm lens.

### Stationary UV–Visible Spectroscopy

UV–vis measurements between 190 and 1600 nm were carried out on a PerkinElmer Lambda 950 UV/vis spectrometer. pSPA and pAZO polymers were spin-coated onto fused silica at a speed of 1500 rpm for 5 min, producing a layer thickness of 680 ± 10 nm. Baseline spectra were recorded on blank substrates, and then spectra of the polymer-coated slides were recorded prior to irradiation and after 30, 120, and 300 s of irradiation with 340 nm light from an unfocused LED (Thorlabs M340L4).

### *In situ* UV–Visible Spectroscopy

*In situ* UV–vis measurements in the visible wavelength range were recorded using the same transmission setup detailed in Metasurface Transmission Measurements, but the NIR Yokogawa OSA instrument was replaced with a visible light responsive Ocean Insight Flame-T spectrometer. The chosen acquisition settings were 4 ms integration time, 50 scan averaging, and a boxcar width of 2. Polymer coatings were prepared on glass coverslips with a spin-coating rate of 1500 rpm. Baseline spectra of the glass slide were measured between 400 and 800 nm on an uncoated region of the coverslip. During irradiation, full spectra were saved every 10 s and the optical density of the polymer film at 570 nm was tracked every 250 ms. Irradiation was provided by Thorlabs LEDs outlined in Section 3.3 of the Supporting Information, which were positioned 20 cm from the sample and focused with a 25 mm lens.

### Spatial Patterning of Polymer-Coated Metasurface

Spatial patterning of the metasurface was achieved with the aid of a DMD. Initially the 457 nm emission from a DPSS CW laser (Photontec MBL-F-457) was expanded to a diameter of 8 mm by using a telescope (*f* = 20 mm and *f* = 50 mm). The beam was then incident upon the DMD (Texas Instruments DLPDLCR3310 evaluation module), which was modified to allow access to the DMD array. The patterned light field was collimated (*f* = 200 mm) and focused onto the sample (*f* = 30 mm). The light transmitted through the sample was again collimated (*f* = 25 mm) and focused (*f* = 100 mm) onto a CMOS camera (Thorlabs DCC1645C-HQ) for alignment purposes. A separate white light arm was coupled via a removable mirror mount for white/NIR light imaging. For this a tungsten halogen lamp (Thorlabs SLS301) was coupled via a vis–NIR fiber and collimated using a condenser lens (*f* = 12 mm). A diagram of the apparatus is provided in Figure S4 of the Supporting Information.

### Nuclear Magnetic Resonance (NMR) Spectroscopy

NMR measurements were conducted on a 300 MHz Bruker NMR spectrometer, using CDCl_3_ or CD_2_Cl_2_ as solvent at 298 K. Chemical shifts are given in parts per million (ppm, δ scale) relative to the residual signals of the deuterated solvents.

### Size Exclusion Chromatography (SEC)

SEC measurements were performed on a Shimadzu system equipped with an SCL-10A system controller, an LC-10AD pump, and an RID-10A refractive index detector using a solvent mixture containing chloroform (CHCl_3_), triethylamine (TEA), and isopropyl alcohol (i-PrOH) (94:4:2) at a flow rate of 1 mL min^–1^ on a PSS SDV linear M 5 μm column. The system was calibrated using PS (100–100000 g mol^–1^) and PEO (440–44700 g mol^–1^) standards.

## Supporting Information Available

Further details on experimental methods, synthetic procedures, and characterization, ellipsometry measurements, and simulated and measured transmission spectra of polymer-coated metasurfaces (PDF)

## Abbreviations:

AIBN: 2,2-azobis(isobutyronitrile)
AZO: azobenzene
DMD: digital micromirror device
EBL: electron beam lithography
FOM: figure of merit
FWHM: full-width at half-maximum
NMR: nuclear magnetic resonance
pAZO: polymers containing azobenzene pendant groups
pAZO-MS: metasurface coated with polymers containing azobenzene pendant groups
pSPA: polymers containing spiropyran pendant groups
pSPA-MS: metasurface coated with polymers containing spiropyran pendant groups
quasi-BIC: quasi-bound states in the continuum
RIU: refractive index unit
SEM: scanning electron micrograph
SPA: spiropyran
UV–vis: UV–visible spectroscopy
