# Supplementary material for: Two-Color Spatially Resolved Tuning of Polymer-Coated Metasurfaces
Source: ACS Nano. 2024 Jan 30;18(6):5079–88. doi: 10.1021/acsnano.3c11760 (PMC10867891; doi:10.1021/acsnano.3c11760)
Supplement: Supplementary file 1 — nn3c11760_si_001.pdf [file nn3c11760_si_001.pdf]

Supporting Information

for

Two-Colour Spatially Resolved Tuning of Polymer Coated Metasurfaces

Sarah L Walden[1,2], Purushottam Poudel[3,4,5], Chengjun Zou[1,2,6], Katsuya Tanaka[1,2], Pallabi Paul[2], Adriana Szeghalmi[2,7], Thomas Siefke[2,7], Thomas Pertsch[2,7], Felix H. Schacher[3,4,5], and Isabelle Staude[1,2]

[1]*Institute of Solid State Physics, Abbe Centre of Photonics, Friedrich Schiller University Jena, Helmholtzweg 3, 07743, Jena, Germany.*
[2]*Institute of Applied Physics, Abbe Centre of Photonics, Friedrich Schiller University Jena, Albert-Einstein-Str. 15, 07745, Germany.*
[3]*Jena Centre for Soft Matter (JCSM), Friedrich Schiller University Jena, Philosophenweg 7, 07743 Jena, Germany.*
[4]*Institute of Organic Chemistry and Macromolecular Chemistry, Friedrich Schiller University Jena, Lessing-Str. 8, 07743, Jena, Germany.*
[5]*Center for Energy and Environmental Chemistry, Friedrich Schiller University Jena, Philosophenweg 7, 07743 Jena, Germany*
[6]*Institute of Microelectronics, Chinese Academy of Sciences, Beitucheng West Road 3, Beijing, China*
[7]*Fraunhofer Institute of Applied Optics and Precision Engineering, Albert-Einstein-Str. 7, 07745 Jena, Germany.*

Contents

1 Supplementary Experimental Methods 2

1.1 Polymer Film Preparation 2

1.2 Ellipsometry 2

1.3 Metasurface Transmission Measurements 3

1.4 Spatial Patterning of Polymer-Coated Metasurface 3

2 Synthesis and Characterisation 4

2.1 Materials 4

2.2 Synthesis of 4-Phenylazophenyl Acrylate (AZO) 4

2.3 SPA Monomer Synthesis (SPA) 4

2.4 Synthesis of pAZO and pSPA Homopolymers 6

3 Supplementary Data 6

3.1 Simulations of Polymer-Coated Metasurface Resonance Tuning 6

3.2 Simulations of Polymer Switching 8

3.3 LED Spectral Analysis 8

3.4 Resolution of Spatial Patterning 9

3.5 Metasurface Resonance Switching with Thick Polymer Film 9

3.6 Resonance Tuning with 1:1 pSPA:pAZO Mixture 10

1 Supplementary Experimental Methods

1.1 Polymer Film Preparation

The resulting film thicknesses produced with a spin rate of 1500 rpm are outlined in Table S1. When these films were found to be too thick, the spin rate was increased 3000 rpm (acceleration of 1000 rpm/s) for 5 mins, producing the film thicknesses reported in Table S1.

Table S1. Spin coat parameters and resulting film thickness determined from ellipsometry measurements.

polymer	RPM	Time	Thickness (nm)
pSPA	1500	5 min	675
	3000	5 min	498
pAZO	1500	5 min	689
	3000	5 min	568

Where both polymers were applied to a single metasurface, the pAZO thin film was prepared first to cover half of the metasurface arrays. The pSPA thin film was then prepared covering the second half of the arrays. Figure S1 depicts the typical separation of the polymer films immediately after the spin coating procedure, as well as after UV and red light irradiation.

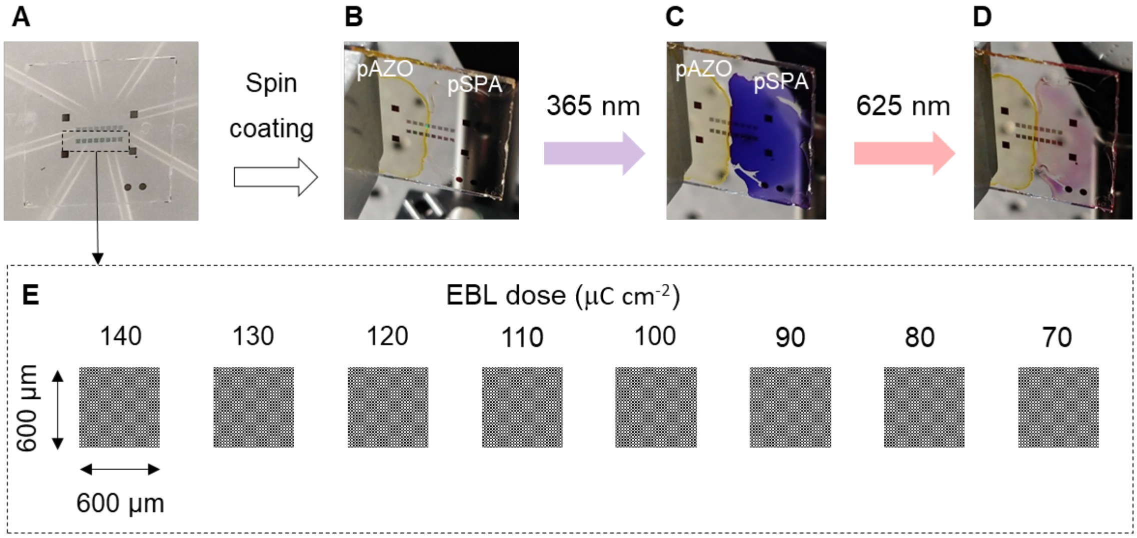

Figure S1. Sample images (A) before polymer coating was applied, (B) after both polymers were applied (note that pSPA is transparent), (C) after irradiation with UV light and (D) after irradiation with red light. (E) Diagram of metasurface arrays with varying EBL doses.

1.2 Ellipsometry

The dispersive refractive indices determined via ellipsometry are shown in Figure S2.

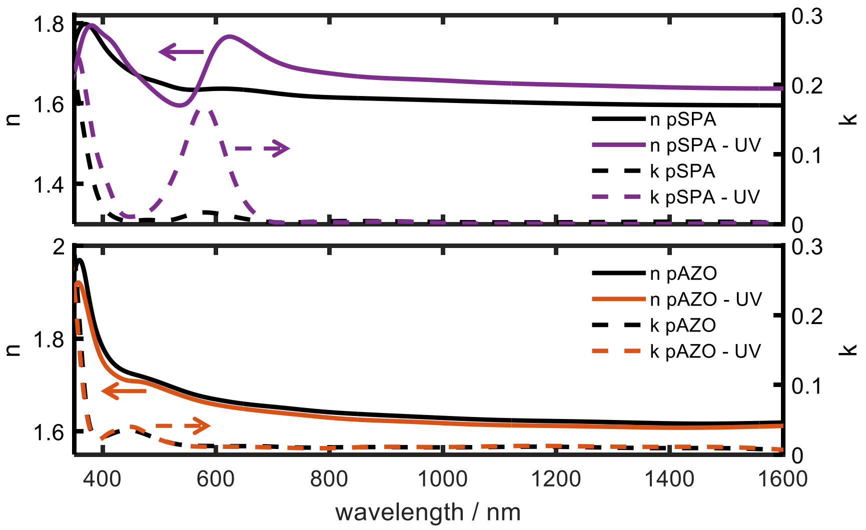

Figure S2. Ellipsometry Data. Full spectrum ellipsometry data of (top) pSPA and (bottom) pAZO, measured before (black) and after (coloured) irradiation with 365 nm LED. Solid lines indicate the real component of the refractive index (left axis) and dashed lines indicate the imaginary component (right axis).

1.3 Metasurface Transmission Measurements

The polarisation dependent transmittance spectra, described in detail in the Experimental Methods section of the manuscript is depicted in Figure S3.

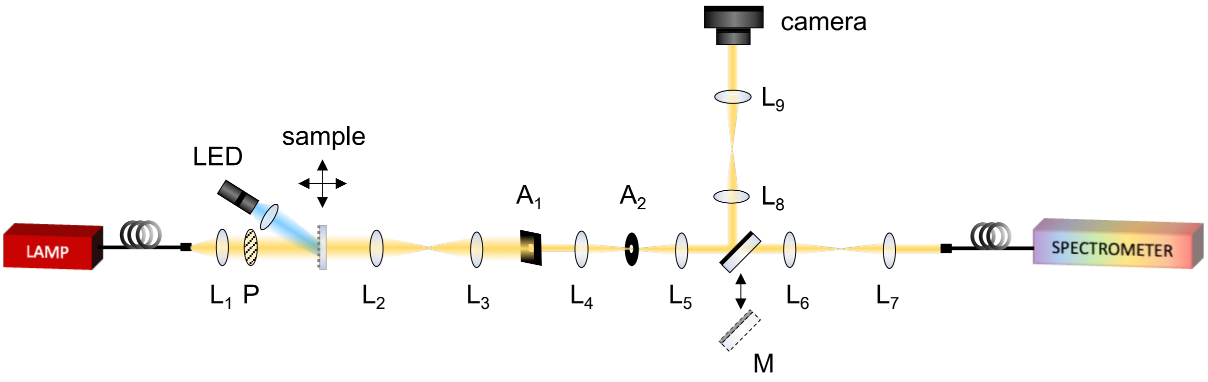

Figure S3. Optical setup for transmittance measurements under UV/blue light exposure. P indicates polarizer, M indicates mirror, A_1 and A_2 and rectangular and circular apertures, respectively. The focal lengths of the marked lenses are: f_{L1} = 7 mm, f_{L2} = 50 mm, f_{L3} = 100 mm, f_{L4} = 50 mm, f_{L5} = 50 mm, f_{L6} = 50 mm, and f_{L7} = 200 mm.

1.4 Spatial Patterning of Polymer-Coated Metasurface

A schematic diagram depicting the apparatus for the spatial patterning of the metasurface using a digital micromirror device (DMD) is depicted in Figure S4. Full experimental details are provided in the Experimental Methods section of the manuscript.

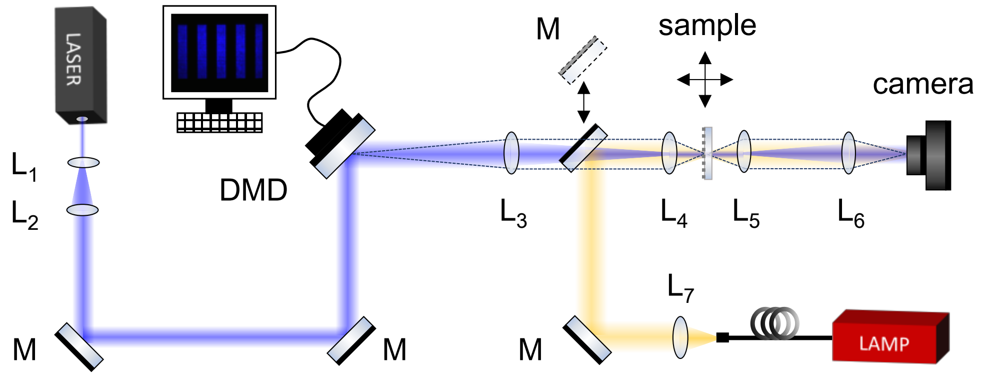

Figure S4. Optical apparatus for spatial patterning of metasurface resonance tuning. Light from a 450 nm CW laser initial passes through beam expansion optics (L_1, f = 20 mm and L_2, f = 50 mm) before being directed onto the DMD. The pattern is then imaged onto the sample and the transmitted light is incident on a CMOS camera. A removable mirror couples in white light for imaging purposes. M indicates a mirror and the focal lengths of the marked lenses are: f_{L1} = 20 mm, f_{L2} = 50 mm, f_{L3} = 200 mm, f_{L4} = 30 mm, f_{L5} = 25 mm, f_{L6} = 100 mm, and f_{L7} = 12 mm.

2 Synthesis and Characterisation

2.1 Materials

4-Phenylazophenol, acryloyl chloride (97.0%), and 2,2-Azobis(isobutyronitrile) (AIBN) were purchased from Sigma-Aldrich (Munich, Germany). 2-Bromoethanol (95.0%), 2,3,3-trimethyl-3H-indol (97.0%), and 2-hydroxy-5-nitrobenzaldehyde (97.0%) were purchased from TCI (Zwijndrecht, Belgium). AIBN was purified through recrystallization from ethanol and stored in the freezer until use. All other reagents were used as received. All the solvents were of analytical grade except 1,4-dioxane and THF, which were purchased from Carl Roth (HPLC grade, Karlsruhe, Germany).

2.2 Synthesis of 4-Phenylazophenyl Acrylate (AZO)

4-Phenylazophenyl acrylate was synthesized as described elsewhere.[1] In short, 4-Phenylazophenol (0.2 mol) and triethylamine (0.26 mol) were dissolved in diethyl ether (200 mL). The solution was purged with nitrogen and cooled to 0 °C. Acryloyl chloride (0.24 mol) dissolved in 80 mL of diethyl ether was then added dropwise to this solution with stirring. The reaction mixture was allowed to reach room temperature and stirred for 8 hours. The reaction solution was filtered to remove triethylammonium salt, washed with water, and evaporated. The product was recrystallized twice from an ethanol-water mixture (3:1) and dried under vacuum. The structure was confirmed by ^{1}H-NMR (Bruker, 300 MHz, CDCl3). δ (ppm) = 6.0 – 6.9 (m, 3H), 7.3 – 7.7(m, 5H), 7.8 – 8.1 (m, 4H).

2.3 SPA Monomer Synthesis (SPA)

2-(3',3'-Dimethyl-6-nitrospiro[chromene-2,2'-indolin]-1'-yl)ethyl acrylate (SPA) was synthesized as described elsewhere.[2] The synthesis route of the SPA monomer was accomplished in the following four steps, as shown in Figure S5.

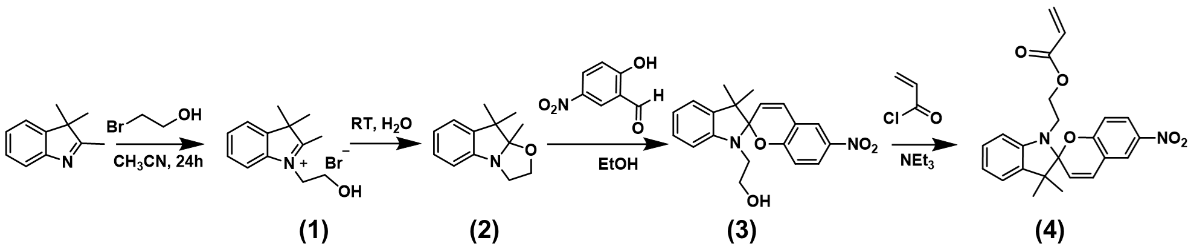

Figure S5. Synthesis route for 2-(3’,3’-Dimethyl-6-nitrospiro[chromene-2,2’-indolin]-1’-yl)ethyl Acrylate (SPA)

(1) Synthesis of 1-(2-Hydroxyethyl)-2,3,3-trimethyl-3H-indolium Bromide

A mixture of 2,3,3-trimethyl-3H-indol (2.72 mL, 16 mmol) and 2-bromoethanol (1.48 mL, 20 mmol, 1.25 eq) in acetonitrile (20 mL) was purged with argon and heated for 26 h under reflux at 100 °C. The solvent was then removed, and the resulting residue was resuspended in 25 mL of hexane. The collected solid was recrystallized from chloroform and used directly in the subsequent step.

(2) Synthesis of 9,9,9a-Trimethyl-2,3,9,9a-tetrahydro-oxazolo[2,3-a] indole

1-(2-Hydroxyethyl)-2,3,3-trimethyl-3H-indolium bromide (step 1) was dissolved in water and mixed with KOH (0.66 g, 12 mmol). The mixture turned from pink to yellow within 10 min at room temperature. The mixture was then extracted with diethyl ether (3 × 20 mL), dried over $MgSO_4$, filtered, and the solvent removed under reduced pressure. The yellow oil (0.8549 g, 33.5%) was analysed via ^{1}H NMR:

^{1}H NMR (300 MHz, CDCl3): δ = 1.89 (s, 3H, CH3), 1.31 (s, 3H, CH3), 1.42 (s, 3H, CH3), 3.44–3.86 (m, 4H, 2 × CH2), 6.76 (d, 1H, CH-arom.), 6.93 (t, 1H, CH-arom.), 7.14 (m, 2H) ppm.

(3) Synthesis of 2-(3’,3’-Dimethyl-6-nitro-3’H-spiro[chromene-2,2’-indol]-1’-yl)-ethanol

9,9,9a-trimethyl-2,3,9,9a-tetrahydro-oxazolo[2,3-a]indole (step 2) (0.85 g, 4,2 mmol, 1 eq) and 2-hydroxy-5-nitrobenzaldehyde (1.054 g, 6.3 mmol, 1.5 eq) were mixed in 10 mL of ethanol and heated under reflux for 3 h. After cooling to room temperature, the remaining solution was filtered and washed with ethanol. The red crystals (0.6319 g, 33%) were analysed via ^{1}H NMR:

^{1}H NMR (300 MHz, DMSO-d6): δ = 1.10 (s, 3H, CH3), 1.20 (s, 3H, CH3), 3.19 (d, 2H, CH2), 3.44 (m, 2H, CH2), 4.72 (t, 1H, OH), 6.01 (d, 1H, CH-arom.), 6.64 (d, 1H, CH-arom.), 6.78 (t, 1H, CH-arom.),6.87 (d, 1H, CH-arom.), 7.11 (m, 3H, 3 × CH-arom.), 8.00 (dd, 1H, CH-arom.), 8.21 (d, 1H, CH-arom.) ppm.

(4) Synthesis of 2-(3’,3’-Dimethyl-6-nitrospiro[chromene-2,2’-indolin]-1’-yl)ethyl Acrylate (SPA)

2-(3’,3’ -Dimethyl-6-nitro-3’H-spiro[chromene-2,2’ -indol]-1’ -yl)-ethanol (step 3) (0.5 g, 1.42 mmol) and trimethylamine (0.197 mL, 1.42 mmol) were dissolved in dichloromethane, purged with argon, and cooled to −35 ◦C. A solution of acryloyl chloride in dichloromethane was added slowly and the reaction mixture was heated up to room temperature overnight. The solution was extracted with saturated $NaHCO_3$ (2 × 20 mL) and water (2 × 20 mL), and the organic phases were combined, dried over $MgSO_4$, filtered, and evacuated under reduced

pressure. The product was purified via column chromatography with chloroform to yield 107.6 mg (0.265 mmol, 18.6%).

^{1}H NMR (300 MHz, DMSO-d6): δ = 1.06 (s, 3H, CH3), 1.19 (s, 3H, CH3), 3.36–3.51 (m, 2 × 1H, CH2), 4.16–4.35 (m, 2 × 1H, CH2), 5.90 (d, 1H, CH2) 5.96 (d, 1H, CH-arom.), 6.09 (dd, 1H, CH), 6.26 (d, 1H, CH2), 6.72 (d, 1H, CH-arom.), 6.80 (t, 1H, CH-arom.), 6.85 (d, 1H, CH-arom.), 7.12 (m, 2H, 2 × CH-arom.), 7.20 (d, 1H, CH-arom.), 7.99 (dd, 1H, CH-arom.), 8.21 (d, 1H, CH-arom.) ppm.

2.4 Synthesis of pAZO and pSPA Homopolymers

pAZO and pSPA were synthesized as follows: 1.0 g of AZO (or SPA) was dissolved in 5 mL 1,4-dioxane. Then 50 mg (5 wt %, 3.04×10^{-4} mol) of 2,2-azobisisobutyronitrile (AIBN) was added, and the mixture was heated at 70 °C for 18 h under argon. The reaction mixture of pAZO (or pSPA) was then precipitated three times in cold hexane and dried at 70 °C for 24 h. The resulting polymers were analysed via SEC and ^{1}H-NMR.

pAZO: ^{1}H-NMR (300 MHz, CD2Cl2): δ =8.0-7.0 (Ar–H) and SEC ($CHCl_3$): M_n = 6500 g/mol, and PDI = 2.5.

pSPA: ^{1}H-NMR (300 MHz, CD2Cl2): δ =8.0-7.6 (Ar–H) and SEC ($CHCl_3$): M_n = 5500 g/mol, and PDI =1.91.

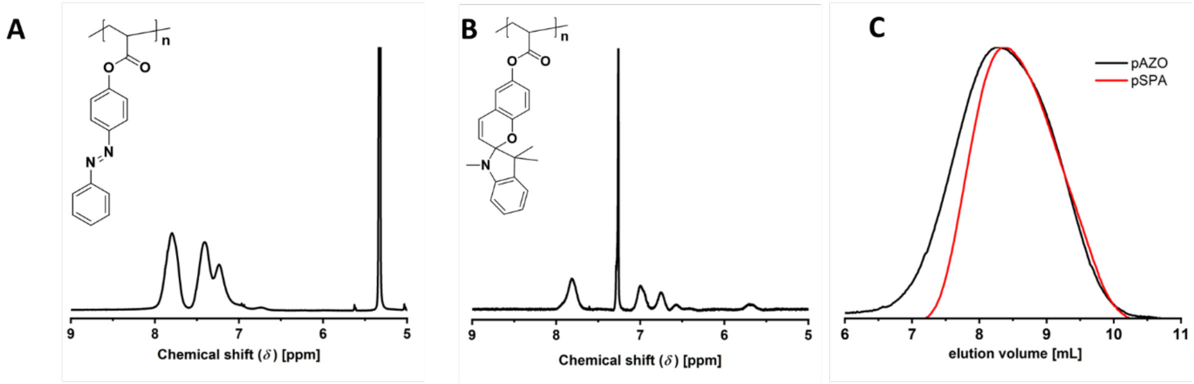

Figure S6: NMR spectra and SEC elution traces: (A) pAZO (in CD_2Cl_2). (B) pSPA (in $CDCl_3$). (C) Normalized SEC traces of pAZO (black) and pSPA (red) using $CHCl_3$ as eluent (PS calibration).

3 Supplementary Data

3.1 Simulations of Polymer-Coated Metasurface Resonance Tuning

For all simulations, the two bar metasurface depicted in Figure 1 of the manuscript is employed. The relevant dimensions are period of unit cell Λ = 770 nm, centre-to-centre gap between resonators G = 360 nm, length of long bar L_1 = 405 nm, length of short bar L_2 = 373 nm, width of both bars W = 242 nm and height of both bars H_{bar} = 270 nm. A full discussion of the influence of these parameters on the resonance positions can be found in our earlier work.[3]

The calculated transmission spectra of the metasurface when coated with 500 nm polymer layer with refractive index varying from 1.0 to 1.7 are presented in Figure S7. The top row presents data for x-polarised incident light and the bottom row is for y-polarised incident light. In both

cases, the shifts of the four main resonances are indicated with a dashed grey line. For x-polarised light, the most significant shift is observed for X3, the anti-parallel electric dipole depicted in the near field profiles of Figure S7b and S7c. This transmission dip associated with resonance X3 shifts from λ = 1235 nm when n = 1.0 to λ = 1483 nm when n = 1.7, (average sensitivity of 354 nm RIU^{-1}) highlighting the sensitivity of this resonance to the local environment. The resonances X1 and X4, arise from the in-plane anti-parallel electric and magnetic dipoles, respectively. Both these resonances are highly localised within the nanobars, and hence only experience a weak shift in response to the refractive index change. Similarly, the transmission dip attributed to X2 arises from the parallel-out of plane electric dipoles located within the nanobars, which are largely insensitive to the polymer refractive index change.

The bottom row of Figure S7, shows the transmission of y-polarised light through the polymer coated metasurface. The main resonance of interest to this work is the parallel out-of-plane magnetic dipole resonance Y3 located between the two bars of the unit cell. The transmission dip attributed to this resonance undergoes a significant shift from λ = 1313 nm when n = 1.0 to λ = 1644 nm when n = 1.7, corresponding to an average sensitivity of 473 nm RIU^{-1}, significantly larger than that of resonance X3. The Q factor of this resonance also increases as the refractive index increases, and the resonance becomes localised between the nanobars. Resonance Y1 is physically the equivalent to resonance Y3, but is located between the nanobars of neighbouring unit cells, as opposed to between the two nanobars within a single unit cell. As a result, this resonance also undergoes a significant shift with refractive index change. Resonance Y2 and Y4 are anti-parallel in-plane and out-of-plane magnetic dipole resonances, respectively. These resonances are both highly localised within the nanobars and hence show minimal shift with refractive index change.

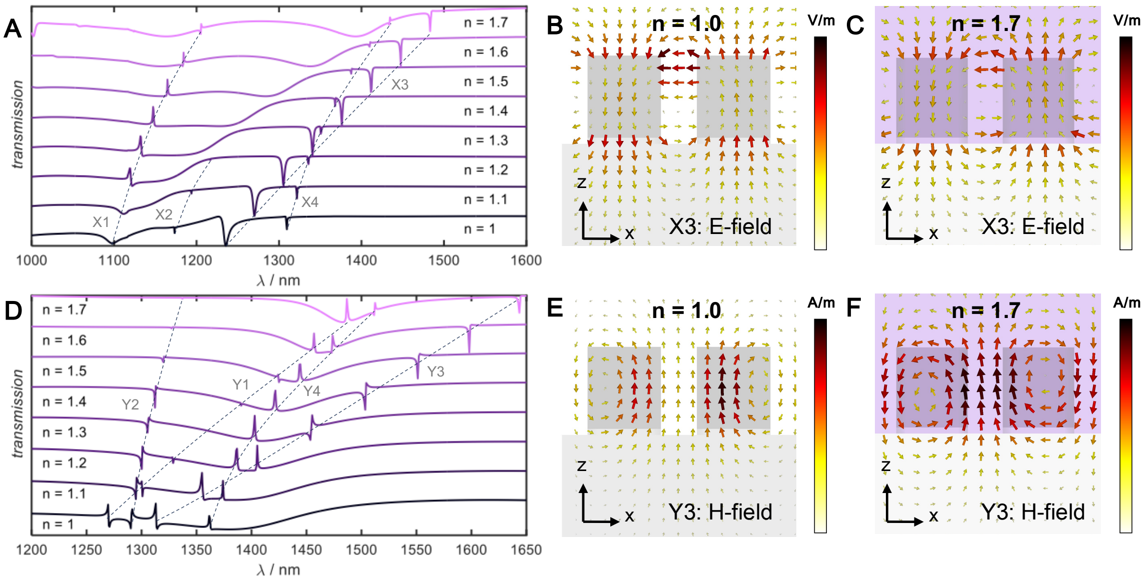

Figure S7. Polymer-coated metasurface simulations. (A,D) simulations showing expected electromagnetic resonances when the two bar metasurface is coated with a 500 nm polymer layer with varying refractive index and illuminated with (A) x- and (D) y-polarised light. Grey dashed lines are a guide to the eye only, highlighting the shift of each resonance. (B,E) Electric near-field profiles and (E,F) magnetic near-field profiles in the unit cell of the metasurface when coated with a polymer of refractive index (B,E) n=1 and (C,F) n=1.7.

3.2 Simulations of Polymer Switching

The expected resonance shifts caused by photoswitch isomerisation, simulated in CST Studio Suite, are presented in Figure S8. The metasurface parameters were identical to those outlined in Section 3.1. The polymer films were assumed to be 500 nm thick, with refractive indices determined from ellipsometry measurements reported in Section 1.3. The top row of Figure S8 contains the data from pAZO coated metasurface (pAZO-MS) and the bottom row presents data from pSPA coated metasurface (pSPA-MS).

For pAZO-MS, the simulations predict a weak blue shift in the resonance position, qualitatively confirming the experimental results. The transmission dips seen here are weaker than those observed in experiments due to small variations in the k values obtained from fits to ellipsometry data. Forcing k to be 0 for $\lambda > 600$ nm increased the magnitude of this dip. For pSPA a strong redshift in the resonance is observed upon switching. The magnitude of the resonance shift is approximately equal to that observed in experiments.

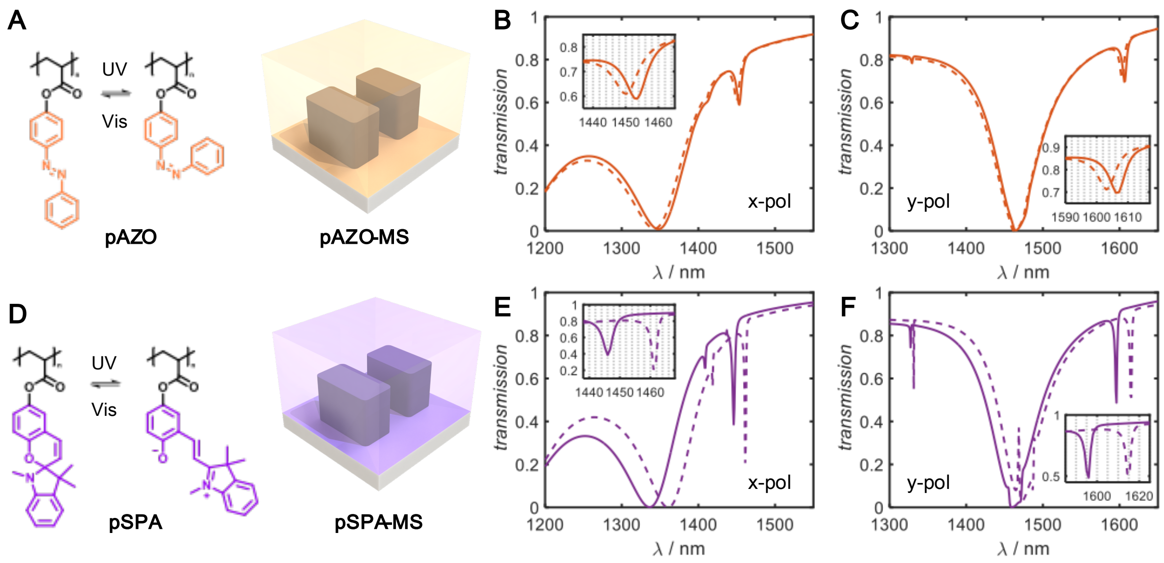

Figure S8. Simulated polymer-coated metasurface tuning. (A,D) structures of photoswitch homopolymers including diagram of polymer coated two bar metasurface. (B-F) Simulations of metasurface resonance position before (solid) and after (dashed) irradiation with UV light, measured with (B,E) x-polarised light and (C,F) y-polarised incident light. (B-C) pAZO coated metasurface, (E-F) pSPA coated metasurface. The insets in (B-F) show close-ups of the transmission within the spectral tuning range of the respective high-Q resonances.

3.3 LED Spectral Analysis

The LEDs used in this work are Thorlabs M340L4, M365L3, M415L4, M625L4, which were all driven by a LED driver Thorlabs LEDD1B. An additional 450 nm (22 cd) from Roithner (Vienna, Austria) was powered by an external power supply. LED emission spectra were recorded using a ocean Insight Flame-T spectrometer and are presented in Figure S9.

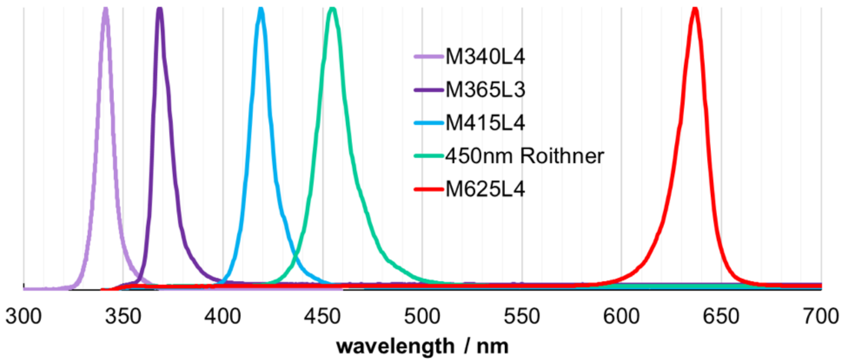

Figure S9. Experimentally recorded spectra of LEDs used in this work.

3.4 Resolution of Spatial Patterning

The resolution of the spatial patterning was assessed by generating periodic patterned 450 nm irradiation using the DMD. First the image size on the camera was calibrated using white light illumination of a single metasurface array with known dimensions of 600 x 600 µm.

A thin film of pSPA was coated onto a glass coverslip using the standard spin coating procedure and was placed into the optical setup outlined in Section 1.7. The entire sample was exposed to 365 nm UV irradiation for 1 minute, and then subsequently exposed to the periodically patterned 450 nm light for 5 minutes. The spatial resolution was determined based on the transmission of the white light arm through the pSPA film (in its initial state SPA will transmit light and after UV irradiation it becomes opaque in the visible region). Based on this analysis the minimum feature resolution was found to be 11 µm. The spatial resolution reported here was limited by the optical setup and choice of objective, and is independent of the properties of the polymer. It is expected that higher resolutions can be achieved with a higher NA objective in place of L_3 in Figure S4.

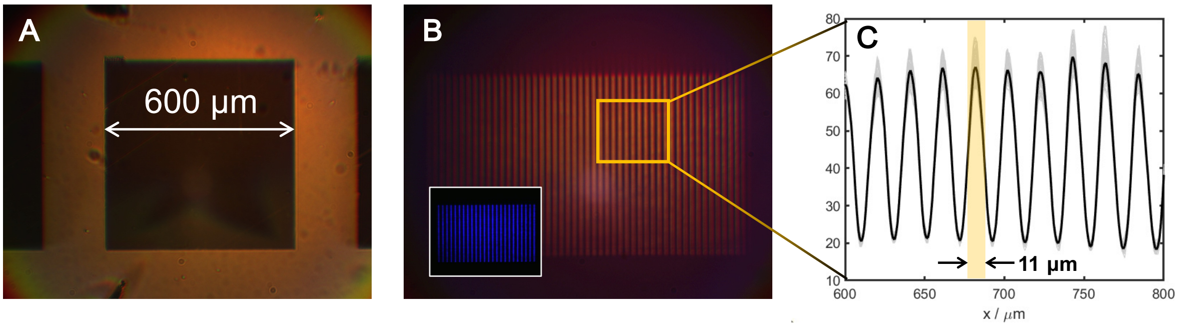

Figure S10: Resolution of spatial patterning. (A) White light image of metasurface array with known dimensions of 600 x 600 µm used to calibrate the scale of the image. (B) white light image of periodic pattern projected onto pSPA film. Inset shows 450 nm DMD projection. (C) greyscale image analysis showing resolution of irradiated lines.

3.5 Metasurface Resonance Switching with Thick Polymer Film

Figure S11 summarises the initial attempts to achieve metasurface resonance tuning with polymer layers spin coated at 1500 rpm (resulting in polymer coatings 680 ± 10 nm thick). Transmission measurements of the polymer-coated metasurface arrays, under various irradiation conditions, are presented in Figure S11a. For ease of interpretation, the same data is presented in Figure S11c normalised to the wavelength of the initial resonance minima.

Further, Gaussian fits were performed to the transmission valleys, so the shift in the minimum wavelength can be easily visualised in Figure S11d.

UV irradiation with a 365 nm LED induced only minor red- and blueshifts of ~1 nm for both the pSPA coated metasurface (pSPA-MS) and pAZO coated metasurface (pAZO-MS), respectively. 5 minutes of 625 nm red light irradiation recovered the pSPA-MS resonance position, but also weakly shifted the pAZO resonance towards its' initial position. 415 nm blue light had the most significant impact, recovering the pAZO-MS resonance position within 2 minutes, but simultaneously caused a significant redshift in the pSPA-MS resonance position. It was this observation that helped to identify the polymer thickness as the primary cause of the weak shifts. Since the light absorption of pSPA at 415 nm is much weaker than at 365 nm (see Figure 2a of the manuscript), better light penetration, and hence switching, is produced deep into the polymer film where the metasurface is located. At 365 nm, the absorption coefficient of the pSPA film is ~6 μm^{-1}, meaning 90% of the light is attenuated before reaching the top of the nanoresonators located 400 nm below the polymer surface. At 415 nm the absorption coefficient reduces to 0.1 allowing 87% of the incident light to penetrate through to the nanoresonators. Reducing the height of the polymer layer, by increasing the speed of the spin coating procedure, while changing no other parameters produced the results presented in Figure 5 of the manuscript.

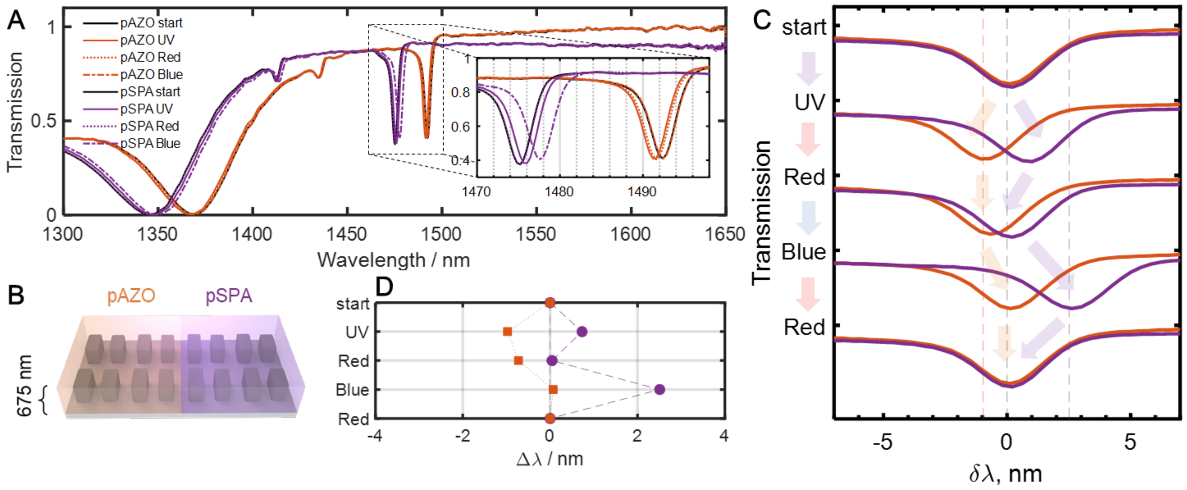

Figure S11. Metasurface resonance tuning with thick polymer film. (A) Transmission measurements of x-polarised light incident on pSPA-MS (purple) and pAZO-MS (orange) measured prior to irradiation (black solid), in the areas exposed to UV light only (solid coloured), red light (dot coloured) and finally blue light (dash-dot coloured). The inset shows a close-up of the transmission within the spectral tuning range of the X3 resonances. (B) schematic diagram of metasurface unit cell indicating height of polymer layer. (C) Metasurface resonance shifts after various irradiation conditions. Data here is the same as in (A) but with the wavelength normalised to the position of the initial resonance minima. (D) Shift in wavelength minima, determined from Gaussian fits to data in (C), after various irradiation conditions.

3.6 Resonance Tuning with 1:1 pSPA:pAZO Mixture

Initally it was planned to spincoat the metasurface with a polymer film containing a 1:1 weight ratio of pSPA and pAZO. For this, separate 5 % wt. stock solutions of pSPA and pAZO were

prepared in CHCl3 and THF, respectively and passed through a 200 µm PTFE filter. 300 µL from each stock solution was added to a fresh vial, which was subsequently placed in an ultrasonic bath for 5 minutes. The absorption properties and switching rates of thin films of each photoswitches prepared with $CHCl_3$ or THF were measured and found to be comparable, as seen in Figure S11. It was therefore deemed that the effect of mixing solvents on the switching behaviour was negligible.

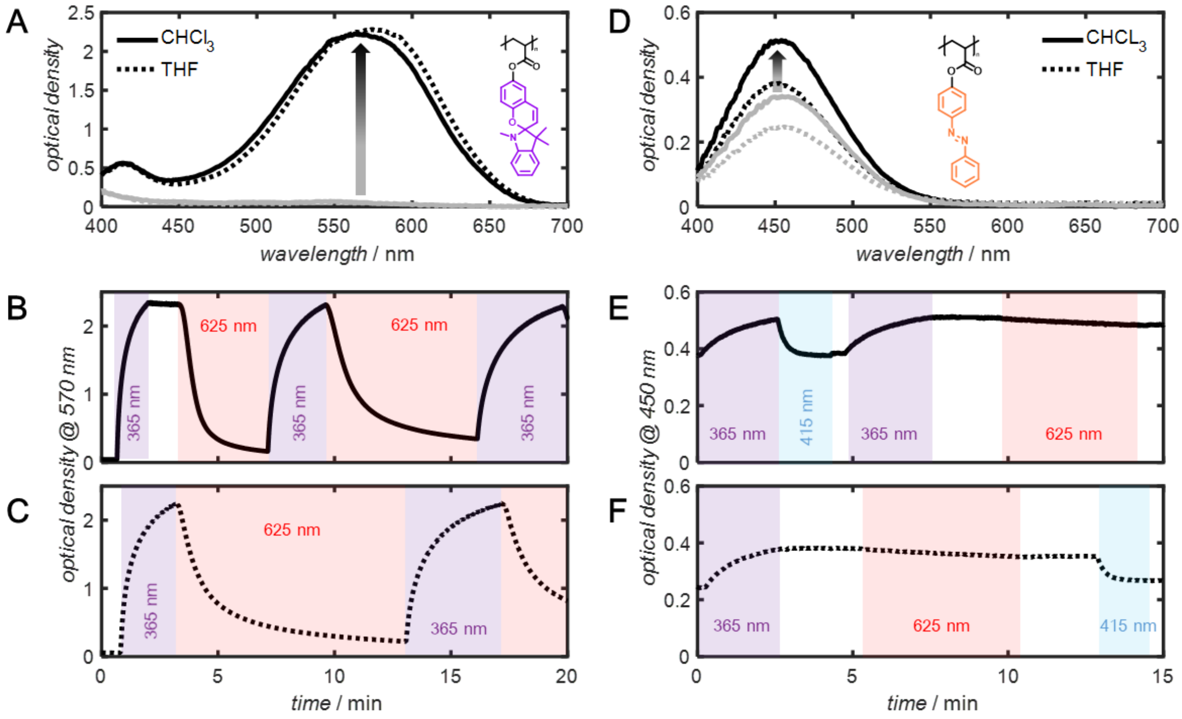

Figure S11: Solvent dependent photoswitching. (A) Optical density spectra of a thin film of pSPA dispersed in THF (solid) and $CHCL_3$ (dotted) before (grey) and after (black) irradiation with a UV LED. Inset shows the chemical structure of pSPA. Arrow indicates wavelength used for kinetic measurements. (B) kinetic optical density measurements of pSPA prepared in $CHCl_3$ at 570 nm during irradiation with various coloured LEDs indicated by shaded regions. (C) same as B, but for pSPA prepared in THF. (D-E) same as A-C, but for pAZO thin films.

A thin film of the polymer mixture was prepared on the metasurface array using the usual spin coating procedure at 1500 rpm. Transmission measurements of the polymer coated metasurface with both x- and y-polarised incident light were performed and are presented in Figure S12. UV light induced an 11 nm and 9 nm redshift in the X3 and Y3 resonances, respectively. No irradiation conditions were identified which produced a significant blueshift from the original resonance position.

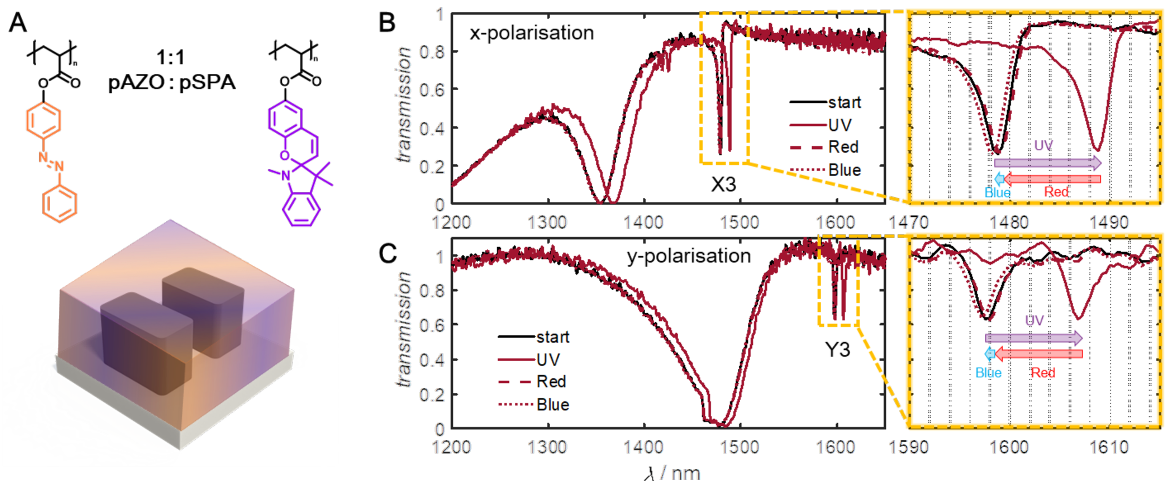

Figure S12: Metasurface resonance tuning with 1:1 polymer mixture. (A) chemical structures and schematic diagram indicating polymer mixture spin coat onto metasurface array. (B-C) Transmission measurements of (B) x-polarised and (C) y-polarised light incident on the metasurface array coated with a 1:1 polymer mixture. Measurements were recorded prior to irradiation (solid black), and after irradiation with UV (solid maroon), Red (dashed maroon) and Blue (dotted marron) LEDs. Insets show zoomed-in region of X3 and Y3 resonances. The insets in (B-C) show close-ups of the transmission within the spectral tuning range of the respective high-Q resonances.

References

(1) Shimoboji, T.; Ding, Z. L.; Stayton, P. S.; Hoffman, A. S. Photoswitching of Ligand Association with a Photoresponsive Polymer−Protein Conjugate. *Bioconjugate Chem.* **2002**, *13* (5), 915–919. https://doi.org/10.1021/bc010057q.

(2) Grimm, O.; Schacher, F. H. Dual Stimuli-Responsive P(NIPAAm-Co-SPA) Copolymers: Synthesis and Response in Solution and in Films. *Polymers* **2018**, *10* (6), 645. https://doi.org/10.3390/polym10060645.

(3) Zou, C.; Poudel, P.; Walden, S. L.; Tanaka, K.; Minovich, A.; Pertsch, T.; Schacher, F. H.; Staude, I. Multiresponsive Dielectric Metasurfaces Based on Dual Light- and Temperature-Responsive Copolymers. *Advanced Optical Materials* **2023**, *11*, 2202187. https://doi.org/10.1002/adom.202202187.
